# NmSEER V2.0: a prediction tool for 2′-O-methylation sites based on random forest and multi-encoding combination

**DOI:** 10.1186/s12859-019-3265-8

**Published:** 2019-12-24

**Authors:** Yiran Zhou, Qinghua Cui, Yuan Zhou

**Affiliations:** 1 Department of Biomedical Informatics, Department of Physiology and Pathophysiology, Center for Noncoding RNA Medicine, MOE Key Lab of Cardiovascular Sciences, School of Basic Medical Sciences, Peking University, 38 Xueyuan Rd, Beijing, 100191 China; 2 Center of Bioinformatics, Key Laboratory for Neuro-Information of Ministry of Education, School of Life Science and Technology, University of Electronic Science and Technology of China, Chengdu, 610054 China

**Keywords:** 2′-O-methylation, Nm site, Random forest, RNA modification, Functional site prediction

## Abstract

**Background:**

2′-O-methylation (2′-O-me or Nm) is a post-transcriptional RNA methylation modified at 2′-hydroxy, which is common in mRNAs and various non-coding RNAs. Previous studies revealed the significance of Nm in multiple biological processes. With Nm getting more and more attention, a revolutionary technique termed Nm-seq, was developed to profile Nm sites mainly in mRNA with single nucleotide resolution and high sensitivity. In a recent work, supported by the Nm-seq data, we have reported a method in silico for predicting Nm sites, which relies on nucleotide sequence information, and established an online server named NmSEER. More recently, a more confident dataset produced by refined Nm-seq was available. Therefore, in this work, we redesigned the prediction model to achieve a more robust performance on the new data.

**Results:**

We redesigned the prediction model from two perspectives, including machine learning algorithm and multi-encoding scheme combination. With optimization by 5-fold cross-validation tests and evaluation by independent test respectively, random forest was selected as the most robust algorithm. Meanwhile, one-hot encoding, together with position-specific dinucleotide sequence profile and K-nucleotide frequency encoding were collectively applied to build the final predictor.

**Conclusions:**

The predictor of updated version, named NmSEER V2.0, achieves an accurate prediction performance (AUROC = 0.862) and has been settled into a brand-new server, which is available at http://www.rnanut.net/nmseer-v2/ for free.

## Background

With the soaring development of genomics and molecular biology, numerous researches have been revealing the pivotal regulatory functions of a great variety of RNA modifications [1].

Among 163 kinds of known post-transcriptional RNA modifications [2], 2′-O-methylation (2′-O-me or Nm), which frequently occurs in ncRNAs and mRNAs, is a peculiar methylation modified at 2′-hydroxy of ribose moiety. Standalone methyltransferases and C/D-box small nucleolar RNA guided enzyme fibrillarin dominate two major ways of Nm modification [3, 4]. Nm modified at specific sites can make contributions to the biogenesis and specificity of rRNA [5, 6], the normal functioning of tRNA [7], the protection effect towards mRNA against degradation by DXO [8] and so on. Driven by the essential functions of Nm, a lot of biochemistry approaches had been designed to detect Nm sites in ncRNAs [9]. Recently, Dai et al. invented a sensitive high-throughput experimental method termed Nm-seq which is capable to detect Nm sites at low stoichiometry especially in mRNAs with single-nucleotide resolution, achieving an unprecedented breakthrough [10].

However, experimental methods are inevitably costly and labor-exhausting. By contrast, prediction approaches in silico seem more efficient and convenient. Along with the explosion of experimental data, prediction algorithms and bioinformatics methods towards large-scale biomedical problems, such as protein-protein interaction prediction [11–18], protein structure analysis [19], eQTL mapping [20] and so on [21–23], had been developed for the past few years. Reviewing through previous studies, functional sites prediction, as a blooming sub-field of bioinformatics, have highlighted many successful application of sequence-based machine-learning prediction framework [24–28], in which the sequence information around functional sites was widely regarded as an easily accessible and powerful tool to extract informative features of functional sites. In a recent work, our group also established a computational prediction tool named NmSEER [29], which was based on the original Nm-seq data (which depicted Nm sites across abundant mRNA and a few ncRNA molecules in HeLa and HEK293 cells’ transcriptome) and random forest (RF) machine learning frame. For clarity, the previous version of NmSEER will be renamed as NmSEER V1.0 hereafter.

NmSEER V1.0 adopted simple one-hot encoding and achieved a decent performance on the original Nm-seq data. However, Dai et al. lately extensively refined the Nm-seq technique and a more credible dataset became recently available. To deal with the much more complicated sequence pattern of Nm sites in this new Nm-seq dataset, a more robust predictor was in urgent demand. For this purpose, we updated our predictor to NmSEER V2.0 by means of not only utilizing the new dataset (Additional file 1: Table S1 and S2), but also adopting multiple sequence encoding strategies and more comprehensive optimization of the classifier.

## Results and discussion

### Comparison among multi-algorithms and multi-encodings

After constructing the training set and testing set from the new Nm-seq data (see ‘Datasets’ subsection), we introduced five single encodings, including one-hot, PSNSP, PSDSP, KNF and KSNPF [30], into this work (see ‘Feature encoding schemes’ subsection). In consideration of the successful application of RF in NmSEER V1.0, we optimized the window size *W* for all single encodings under RF via 5-fold cross-validation tests on the training set (positive-to-negative ratio of 1:10 unless otherwise stated). As results, *W-*values of one-hot, PSNSP, PSDSP, KNF and KSNPF were finally determined as 10, 16, 15, 16 and 5, respectively.

In order to investigate the best algorithm compatible with these five encodings, we trained several models based on the same training set but different machine learning algorithms (seven algorithms in total, see ‘Machine learning algorithms’ subsection) for each encoding. After optimization of necessary hyper-parameters, we rigorously performed independent test on the testing set for all models. The comparison of area under receiver operating characteristic curve (AUROC) and area under precision-recall curve (AUPRC) among seven algorithms based on various encodings are listed in Tables 1 and 2, respectively. Due to the extremely imbalanced positive-to-negative ratio of our independent testing set (1:50, see ‘Datasets’ subsection for more details), the AUPRC are much lower than AUROC (since precision will drop much more sharply in such extreme imbalanced dataset than specificity). Nevertheless, the overall performance of most ‘algorithm + encoding combinations’ is acceptable, showing an AUROC > 0.8 and AUPRC > 0.1 in such extreme situation. The results also suggest the best accuracy of RF for most cases, followed by MLP, CNN and SVM. By contrast, LR, Adaboost and GaussianNB showed uneventful performance only. One challenging issue for these machine learning algorithms is the imbalanced positive-to-negative ratio of our training set (1:10). Not all machine learning algorithms are robust to the imbalanced ratio. However, RF overcome the challenge and RF models trained on the imbalanced training dataset even performed better than that trained on the balanced training dataset (Tables 1 and 2). Therefore, RF was chosen as the most robust algorithm to build our NmSEER v2.0 predictor.

**Table 1:** AUROC comparison among seven algorithms based on multi-encodings by independent test

Algorithm\Encoding	One-hot	PSNSP	PSDSP	KNF	KSNPF
RF	0.811	0.804	0.850	0.761	0.722
LR	0.761	0.762	0.833	0.761	0.740
GaussianNB	0.756	0.753	0.824	0.729	0.646
Adaboost	0.756	0.765	0.847	0.747	0.704
SVM	0.810	0.747	0.831	0.686	0.730
MLP	0.810	0.777	0.844	0.758	0.759
CNN	0.822	0.771	0.842	0.749	0.653
RF (1:1 training set)	0.801	0.797	0.848	0.763	0.724

**Table 2:** AUPRC comparison among seven algorithms based on multi-encodings by independent test

Algorithm\Encoding	One-hot	PSNSP	PSDSP	KNF	KSNPF
RF	0.191	0.177	0.240	0.102	0.095
LR	0.073	0.077	0.153	0.066	0.067
GaussianNB	0.071	0.075	0.150	0.052	0.032
Adaboost	0.070	0.075	0.192	0.058	0.056
SVM	0.178	0.113	0.178	0.041	0.095
MLP	0.159	0.087	0.226	0.064	0.085
CNN	0.145	0.090	0.189	0.064	0.033
RF (1:1 training set)	0.155	0.149	0.211	0.089	0.096

### Performance improvement by combined encoding schemes

**Fig. 1:**
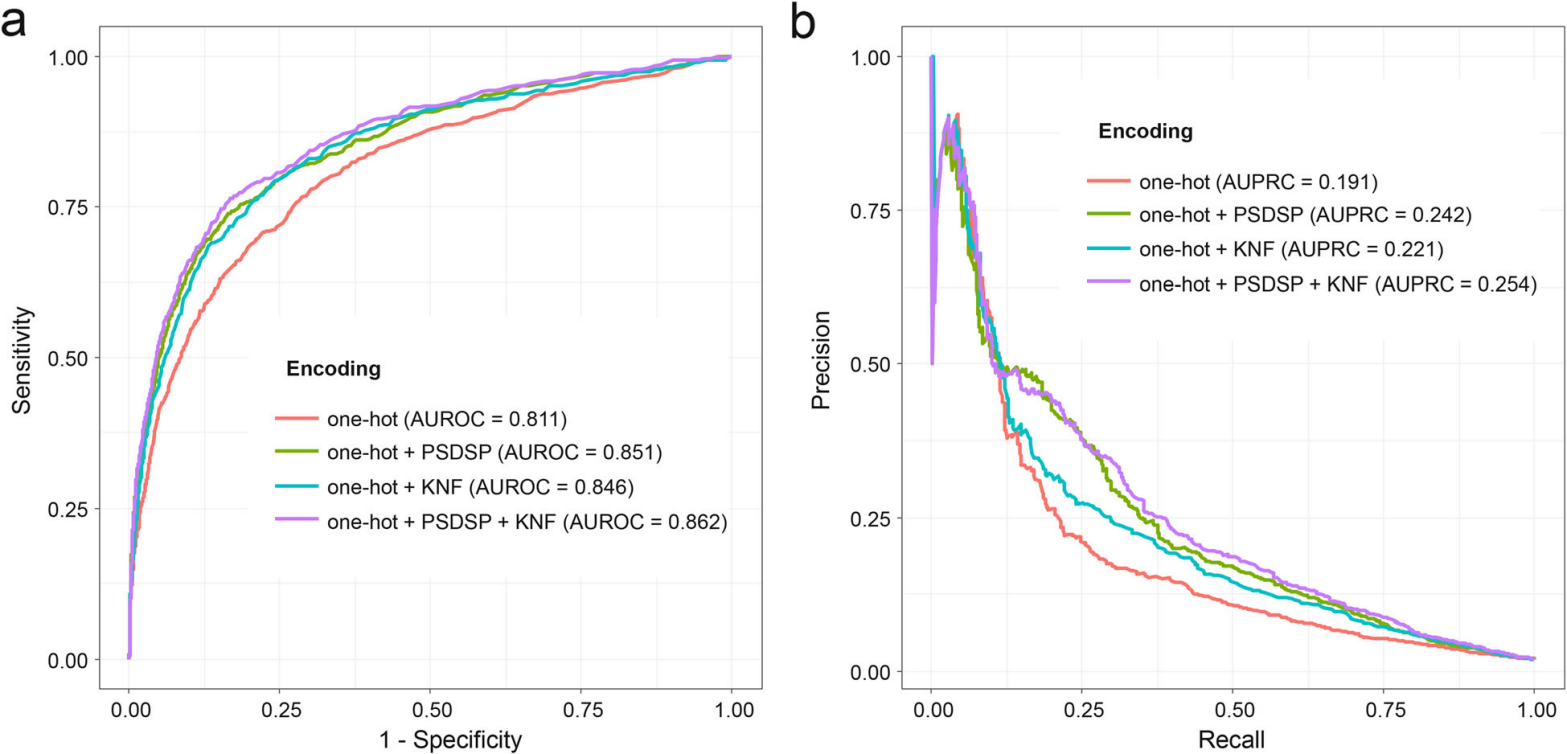
The improvement of RF models based on encoding combinations. **a** ROC curves comparing the performance between predictors using single encoding and encoding combinations, based on the independent test. **b** PRC curves comparing the performance between predictors using single encoding and encoding combinations, based on the independent test

Inspired by the efficiency of single one-hot encoding in profiling nucleotide sequence in NmSEER V1.0, we continued to use one-hot and aimed to achieve better accuracy by combining one-hot with some of the newly-introduced encodings above (i.e., PSNSP, PSDSP, KNF and KSNPF). Distinct to simple one-hot encoding, PSNSP and PSDSP encodings depict the position-specific difference between positive and negative samples, while KNF and KSNPF encodings emphasize on finding the frequency pattern of nucleotides or short sequence motifs. Besides RF’s prominence, Tables 1 and 2 also reflect the much better performance of PSDSP and KNF encodings in profiling Nm sites, comparing with their analogous counterparts, i.e. PSNSP and KSNPF encodings, respectively. Therefore, RF models based on the superior PSDSP and KNF encodings were retained for the further combination with one-hot. In other words, predictors based on three selected encoding combinations, including one-hot + PSDSP, one-hot + KNF and one-hot + PSDSP + KNF, were established. To achieve such encoding combination, the prediction scores from these three models were integrated by weighted sum (see ‘Feature encoding schemes’ subsection). We calculated AUROC and AUPRC for all combinations according to the independent test on the testing set. Figure 1 illustrates performance of them. It is obvious that the combination of one-hot + PSDSP + KNF achieved performance improvement to the largest extent, which implies that positional specific sequence pattern and position-independent nucleotide frequency around Nm sites are both informative in predicting Nm sites. Hence the one-hot + PSDSP + KNF combination was finally determined as the most powerful one.

### Performance of feature selected model

After determining RF and one-hot + PSDSP + KNF as the best algorithm and encoding scheme combination respectively, we were interested in whether feature selection would be helpful to further improve the prediction accuracy (see ‘Feature encoding schemes’ subsection). According to the distribution of importance scores, we extracted top 15 and 50 features from 30-dimensional PSDSP feature vector and 336-dimensional KNF feature vector, respectively. The whole one-hot vector was retained since its feature importance scores did not significantly vary among features. We subsequently constructed the prediction models based on these top features and carried out the same independent test mentioned above. Unfortunately, however, feature selection do not result in improvement of overall performance (with the AUROC of 0.857 and AUPRC of 0.252 after feature selection, versus the AUROC of 0.862 and AUPRC of 0.254 before feature selection). Nevertheless, we still list the top 10 features selected from each encoding in Table 3, which may be valuable to profile and understand the Nm-related sequence motifs. At last, the intact one-hot + PSDSP + KNF encoding combination was employed to build the final prediction model.

**Table 3:** Description of top 10 features from one-hot, PSDSP and KNF encodings

Rank	In one-hot	PSDSP	KNF
1	T at 0 position	Dinucleotide at − 1 and 0 position	Frequency of GA
2	A at −2 position	Dinucleotide at 0 and + 1 position	Frequency of TG
3	C at −3 position	Dinucleotide at −2 and − 1 position	Frequency of AG
4	C at −1 position	Dinucleotide at −3 and − 2 position	Frequency of CT
5	G at −3 position	Dinucleotide at −5 and − 4 position	Frequency of GG
6	G at −1 position	Dinucleotide at −4 and − 3 position	Frequency of AA
7	G at −6 position	Dinucleotide at −9 and − 8 position	Frequency of CC
8	G at −9 position	Dinucleotide at −8 and − 7 position	Frequency of TC
9	G at −8 position	Dinucleotide at −10 and − 9 position	Frequency of CA
10	G at −10 position	Dinucleotide at −6 and − 5 position	Frequency of GC

### NmSEER V2.0 server

According to above results, we appointed the RF model based on one-hot + PSDSP + KNF encoding combination as the final prediction model of NmSEER V2.0. Moreover, we built two specific models for predicting Nm sites in individual HeLa and HEK293 cell type under the same framework, by using the training dataset from each cell line, respectively (corresponding training and testing sets are listed in Additional file 1: Table S3-S6).

**Fig. 2:**
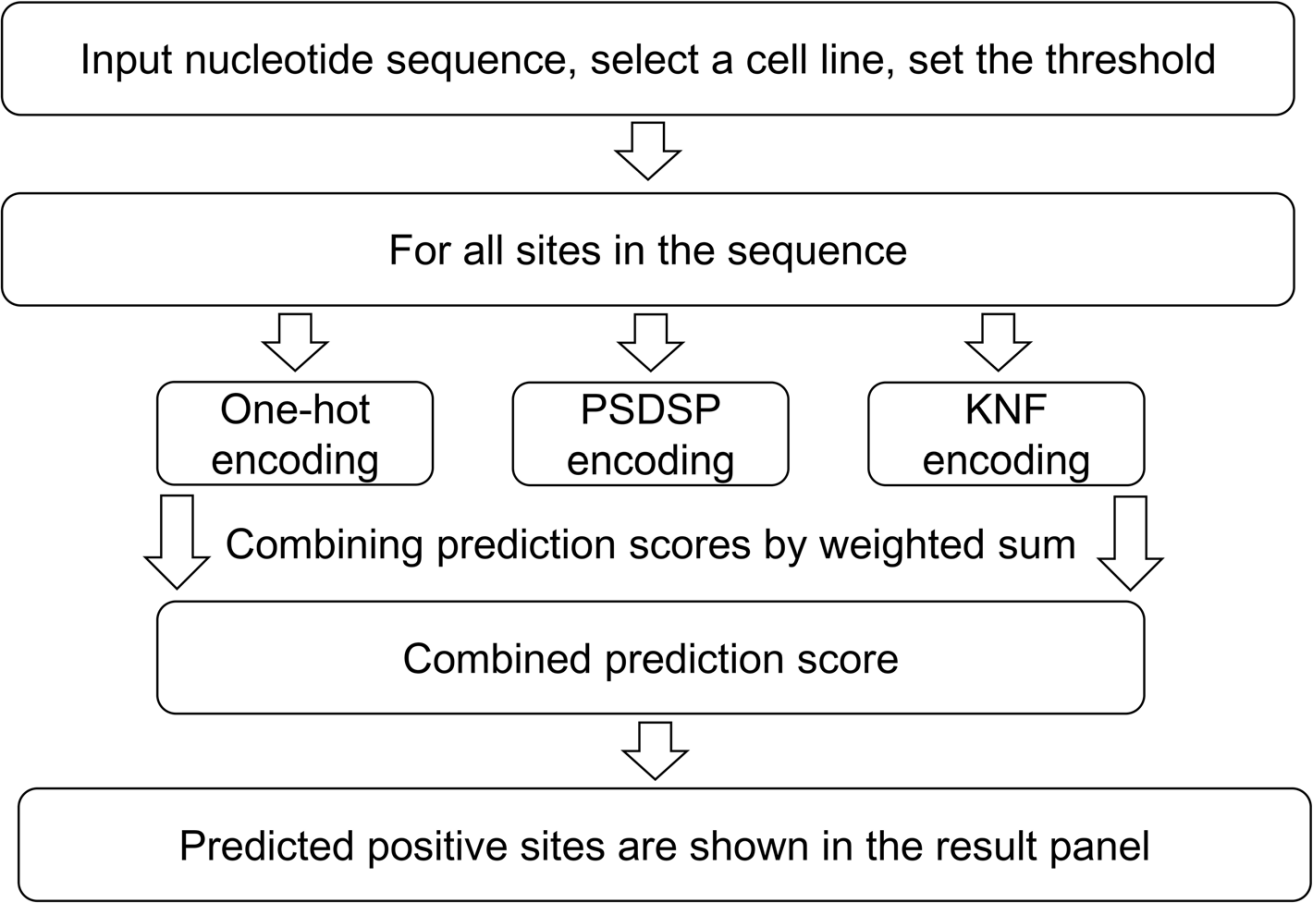
The workflow of NmSEER V2.0 server. Before a task launches, it is necessary to input a nucleotide sequence, select a cell line (by default, Nm sites from both cell lines are considered) and set a stringency threshold for the predictor. Launched task will call the predictor on the server and final classification and prediction scores of sites whose scores higher than the user-selected threshold will be shown in the result panel of webpage

NmSEER V2.0 has been established as a brand-new sever, which is freely available at http://www.rnanut.net/nmseer-v2/. The computational framework of NmSEER V2.0 is illustrated in Fig. 2. For users’ convenience, we provide three pairs of pre-defined thresholds for NmSEER V2.0 server, which correspond to the true positive rate of 0.2, 0.5 and 0.8 in independent test. By default, the predictor trained on the dataset from both cell types is enabled, but the user can easily switch to cell type-specific models on the web interface. Table 4 shows the performance of the default and cell type-specific models of NmSEER V2.0 at each threshold.

**Table 4:** Performance of NmSEER V2.0 at the true positive rate of 0.2, 0.5 and 0.8

Cell Type	True positive rate	Threshold	Specificity	Precision	F_1_-score
Both (default)	0.2	0.338	0.995	0.438	0.274
Both (default)	0.5	0.201	0.955	0.186	0.271
Both (default)	0.8	0.103	0.763	0.064	0.118
HeLa	0.2	0.338	0.994	0.389	0.264
HeLa	0.5	0.205	0.949	0.162	0.244
HeLa	0.8	0.104	0.745	0.059	0.110
HEK293	0.2	0.369	0.997	0.556	0.297
HEK293	0.5	0.250	0.981	0.349	0.411
HEK293	0.8	0.136	0.894	0.132	0.226

## Conclusions

Nm is a widespread post-transcriptional modifications in both ncRNAs and mRNAs and plays important roles in various biological processes. In this study, supported by the new dataset of refined Nm-seq technique, we updated NmSEER to V2.0 by building a new RF model with the enhanced one-hot + PSDSP + KNF encoding combination, which achieves robust prediction performance (AUROC = 0.862 and AUPRC = 0.254) in the independent test. NmSEER V2.0 has been established as a brand-new sever, which is available at http://www.rnanut.net/nmseer-v2/ for free.

## Methods and materials

### Datasets

NmSEER V1.0 was trained with the previous version of Nm-seq dataset [10], which enabled a dataset of positive Nm samples across HeLa’s and HEK293’s genome with single-nucleotide resolution. Nevertheless, Dai et al. refined Nm-seq technology more recently, which resulted in a new credible dataset (GEO Accession: GSE90164) where RefSeq ID of transcripts and positions of Nm sites in transcripts were recorded [10]. We utilized the similar approach in NmSEER V1.0 [29] to generate training set and independent testing set from this brand-new Nm-seq dataset to build NmSEER V2.0. For detail, we merged all the Nm sites of HeLa and HEK293 and mapped them into human transcriptome (Version GRCh38, recorded by RefSeq database) for preparation [31]. Subsequently, positive samples in approximate three fourth of transcripts, which harbor at least one Nm site, were assigned randomly to the training set, and the rest one fourth were assigned to the independent testing set. Because of the absence of golden negative samples from the experimental data, we had to randomly select non-modified RNA sites as negative samples. Since in natural transcripts the amount of non-Nm sites is about 2000-fold more than Nm sites according to our observation, it is not possible to check through all the potential negative samples. Therefore, in the overall consideration of computational efficiency and generalization capability, a positive-to-negative ratio of 1:10 for training set and a more rigorous ratio of 1:50 for testing set were determined for the research. Theoretically, the imbalanced positive-to-negative ratio is capable of evaluating the real-world performance of prediction models objectively. Moreover, for the purpose of emphasizing Nm sites and avoiding the bias to non-Nm sites adjacent to Nm sites, 50% of the negative samples were selected from the proximal region of known Nm sites (i.e. 50 nt flanking windows where the positive samples settled in the center) and the other 50% were sampled from the remaining distal regions. Consequently, we picked 1989 positive samples and 20,025 negative samples from 950 transcripts to assemble the training set, and 657 positive samples and 32,363 negative samples from 328 transcripts to construct the independent testing set (Additional file 1: Table S1 and S2). In addition, to investigate the relationship between prediction performance and positive-to-negative ratio, a subset of training set with positive-to-negative ratio of 1:1 was extracted.

### Machine learning algorithms

We introduced several widely-used machine learning algorithms, including random forest, logistic regression, Naïve Bayes, Adaboost, support vector machine, multilayer perceptron and convolutional neural network, to search for the most suitable model. Except multilayer perceptron and convolutional neural network constructed via python software package *tensorflow*, all the algorithms were implemented by python software package *sklearn* [32], and key hyper-parameters were optimized via 5-fold cross-validation on the training set (Additional file 1: Table S7). Brief introduction of these algorithms will be available below.

#### Random forest (RF)

Random forest model is a meta-classifier which can mature numerous decision trees by learning from the training data, and predict through voting across these trees. NmSEER V1.0 was established under random forest framework.

#### Logistic regression (LR)

LR is a generalized linear model to solve binary classification problems. Mathematically, logistic regression modifies linear regression with a sigmoid function, which leads to the range of (0, 1) that can be regarded as the prediction probability score.

#### Naïve Bayes

Naïve Bayes is a set of traditional machine learning algorithms based on Bayes theorem. In this study, Gaussian Naïve Bayes (GaussianNB) was employed as the representative.

#### Adaboost

The core principle of Adaboost is integrating a number of weak classifiers into a strong classifier, in which a classifier is fitted by the training data first, and then the other copies of classifier focus more on learning from mistakes via adjusting the weights of incorrectly classified instances.

#### Support vector machine (SVM)

SVM is a prominent algorithm towards small sample size, and it is popular in bioinformatics researches [33, 34]. One key technical advantage of SVM is to use kernel function to project low-dimension data into a high-dimension space so that the data will be more distinguishable.

#### Multilayer perceptron (MLP)

MLP is a traditional model of neural network. In this work, we constructed a network model containing 5 hidden layers and a softmax output layer. In view of the instability of neural network optimizer, averaged results from 10 times training were calculated to assess the final prediction performance result,

#### Convolutional neural network (CNN)

CNN is a rising deep learning model with an incomparable performance in classifying image data [35]. Moreover, its effectiveness to predict functional sites have also been proved in recent researches [36, 37]. The CNN model we built here contained 4 convolution layers, a max pooling layer, a flatten layer, 3 full-connected layers and a softmax output layer. The final prediction performance was likewise averaged from 10 times of training and testing.

### Feature encoding schemes

Proper feature encoding scheme plays an extremely important role in modification site prediction. In this study, we attempted to utilize several encodings to translate nucleotide sequences of *W* nt flanking windows on both sides in which sample sites were deployed at center (i.e., 2 × *W* + 1 nt in total) into feature vectors as the input of machine learning models. Flanking windows which were out of the terminus of RNA modules were filled by gap characters. Exact *W*-value of each encoding scheme was optimized via 5-fold cross-validation tests on the training data. Elaborate introduction of encoding schemes will be provided below.

#### One-hot encoding of positional nucleotide sequence (one-hot)

One-hot encoding aims to denote each nucleotide as a 4-dimensional binary vector [29, 38]. In this study, nucleotide A, G, C, T and the gap character were translated as (1, 0, 0, 0), (0, 1, 0, 0), (0, 0, 1, 0), (0, 0, 0, 1) and (0, 0, 0, 0), respectively. Consequently, a nucleotide sequence in *W*nt flanking window corresponds to a 4 × (2 × *W* + 1)-dimensional binary feature vector under the one-hot scheme. NmSEER V1.0 works with only one-hot encoding.

#### Position-specific nucleotide sequence profile (PSNSP)

PSNSP is a prevalent encoding scheme in extracting features from sequence information [30, 39]. It profiles the percentage difference of each nucleotide occurring at each position between positive and negative sequences in *W*nt flanking window. Namely, PSNSP-value could be calculated according to the formula:

1 $$ PSNSP\left(i,n\right)= fp\left(i,n\right)- fn\left(i,n\right) $$

where *i* represents the *i*-th position of sample sequence and *n* generalizes four nucleotides A, G, C or T, then *PSNSP(i,n)* represents the PSNSP-value of nucleotide *n* occurring at position *i*. *fp(i, n)* and *fn(i, n)* are the frequency of nucleotide *n* occurring at position *i* in all positive sequences and negative sequences, respectively. For each site in sample sequences, a PSNSP-value was calculated by formula 1 where corresponding position *i* and nucleotide *n* as the input parameters, and for gap characters out of the terminus of transcripts, 0 was used. Hence a (2 × *W* + 1)-dimensional PSNSP feature vector was generated for each sample.

#### Position-specific dinucleotide sequence profile (PSDSP)

Researches have revealed the effectiveness of PSDSP in functional site prediction [30, 40]**.** Similar to PSNSP, PSDSP encoding calculates the frequency difference of each dinucleotide occurring at each position between positive and negative sequences in *W*nt flanking window to compose feature vectors. 0 was used for gap characters. As a result, each sample was translated into a (2 × *W*)-dimensional PSDSP feature vector.

#### K-nucleotide frequencies (KNF)

As one of the most popular encoding schemes, KNF is skilled in finding polynucleotide patterns by calculating the frequency of all possible *k*-mer polynucleotides in a sequence [25, 27, 30, 41]. Here we used *k* = 2, 3 and 4 to sample sequences in *W*nt flanking window. Therefore, the dimension of KNF feature vector is 4^2^ + 4^3^+ 4^4^ = 336.

#### K-spaced nucleotide pair frequencies (KSNPF)

KSNPF calculates the frequencies of 16 pairs of nucleotides spaced by *k*-length polynucleotides in a sequence [30, 42]. Here we used *k* = 0, 1, 2, 3 and 4 for sample sequences in *W*nt flanking window. Therefore, the dimension of KSNPF feature vector is 5 × 4 × 4 = 80.

#### Weighted encoding combination

If single encodings tend to complement each other, their cooperation will be efficient to improve the prediction performance further, and vice versa. In this work, the encoding combination was implemented by calculating weighted sum of prediction scores from 5-fold cross-validation tests of individual encodings. The weighted sum formula can be described as:

2 $$ Score(s)=\sum \limits_{i=1}^n{w}_i{m}_i(s),\sum \limits_{i=1}^n{w}_i=1 $$

where *Score(s)* indicates the combined prediction score of sample *s*, *n* represents the total number of encodings considered, *w*_*i*_ represents the weight of the *i*-th encoding and *m*_*i*_*(s)* indicates the prediction score of sample *s* based on the *i*-th encoding model. For optimization, the weight of each encoding was tuned from 0 to 1 with the step of 0.01.

#### Feature selection

Unnecessary features in the feature vector sometimes may encumber the performance of prediction model so that selecting informative features may be useful for further performance improvement. According to the Gini impurity decrease, random forest model can provide importance scores of all features. Therefore, referring to the distribution of importance scores, we could reasonably select top informative features from the interested encodings.

### Performance evaluation

Performance was evaluated via independent test on the abovementioned testing set. In independent test, machine learning models based on different algorithms and encoding schemes will output prediction score for each sample, and a sample whose score is higher than an arbitrary threshold will be classified as positive prediction. Hence the count of true positive, true negative, false positive and false negative predictions, which are denoted respectively as *TP*, *TN*, *FP* and *FN*, could be calculated. Then we introduced some typical indicators including *sensitivity*, *specificity*, *recall*, *precision* and *F1-score* to evaluate the predictor’s performance, which are defined as:

3 $$ Sensitivity=\mathrm{Recall}=\frac{TP}{TP+ FN} $$

4 $$ Specificity=\frac{TN}{TN+ FP} $$

5 $$ \mathrm{Precision}=\frac{TP}{TP+ FP} $$

6 $$ {F}_1- score=2\times \frac{Precision\times Recall}{Precision+ Recall} $$

Furthermore, based on the above-mentioned indicators, we plotted receiver operating characteristic curves (ROC curve) and precision-recall curves (PRC curve). ROC curve reveals the relationship between *sensitivity* and 1 – *specificity* under variable thresholds, and PRC curve depicts the tendency of *precision* with *recall* changing. As two golden standards, these curves can visually evaluate the overall performance of prediction models. Especially, PRC curve is suitable for this study because of its stringency on the testing dataset with imbalanced positive-to-negative ratio. To quantify the models’ performance, we calculated the area under ROC and PRC (i.e. AUROC and AUPRC, respectively) as the decisive indicators. For both of them, the value closer to 1 reveals the better prediction performance.

## Supplementary information

**Additional file 1:** **Table S1.** Training set for both HeLa and HEK293. **Table S2.** Testing set for both HeLa and HEK293. **Table S3.** Training set for individual HeLa. **Table S4.** Testing set for individual HeLa. **Table S5.** Training set for individual HEK293. **Table S6.** Testing set for individual HEK293. **Table S7.** Optimized hyper-parameters of each encoding.

## Data Availability

The authors declare that the data supporting the findings of this study are available within the article and its supplementary information files.

## Abbreviations

AUPRC: Area under precision-recall curve
AUROC: Area under receiver operating characteristic curve
CNN: Convolutional neural network
GaussianNB: Gaussian Naïve Bayes
GEO: Gene expression omnibus
KNF: K-nucleotide frequencies
KSNPF: K-spaced nucleotide pair frequencies
LR: Logistic regression
MLP: Multilayer perceptron
Nm: 2′-O-methylation
PSDSP: Position-specific dinucleotide sequence profile
PSNSP: Position-specific nucleotide sequence profile
RBF: Radial basis function
RF: Random forest
SVM: Support vector machine
